# Physical Activity During Pregnancy is Associated with Improved Breastfeeding Outcomes: A Prospective Cohort Study

**DOI:** 10.3390/ijerph16101740

**Published:** 2019-05-16

**Authors:** Phung Thi Hoang Nguyen, Colin W. Binns, Cong Luat Nguyen, Anh Vo Van Ha, Khac Tan Chu, Dat Van Duong, Dung Van Do, Andy H. Lee

**Affiliations:** 1 Faculty of Public Health, University of Medicine and Pharmacy at Ho Chi Minh City, Ho Chi Minh City 700000, Vietnam; phungnh@gmail.com or nthphungytcc@ump.edu.vn (P.T.H.N.); dvdung@ump.edu.vn (D.V.D.); 2 School of Public Health, Curtin University, Perth, WA 6845, Australia; luatcong.nguyen@postgrad.curtin.edu.au (C.L.N.); anhhvv@pnt.edu.vn (A.V.V.H.); cktan@hpmu.edu.vn (K.T.C.); datvanduonghanoi@gmail.com (D.V.D.); Andy.Lee@curtin.edu.au (A.H.L.); 3 National Institute of Hygiene and Epidemiology, Ha Noi 100000, Vietnam; 4 Faculty of Public Health, Pham Ngoc Thach University of Medicine, Ho Chi Minh City 700000, Vietnam; 5 Epidemiology Department, Hai Phong University of Medicine and Pharmacy, Hai Phong City 180000, Vietnam

**Keywords:** breastfeeding, breastfeeding duration, physical activity, pregnancy, prospective cohort, Vietnam

## Abstract

Physical activity is important for health, but little is known about associations between physical activity during pregnancy and breastfeeding. The aim of this study was to investigate any association between antenatal physical activity and breastfeeding duration. A prospective cohort of 2030 Vietnamese women, recruited between 24 and 28 week-gestation was followed up to twelve months postpartum. Physical activity was determined using the pregnancy physical activity questionnaire at baseline interview. Data was available for 1715 participants at 12 months, a 15.5% attrition rate. At 12 months 71.8% of mothers were still breastfeeding. A total of 20.9% women met physical activity targets and those mothers undertaking higher levels of physical activity had a lower risk of breastfeeding cessation by twelve months [hazard ratios HR = 0.59 (95% CI 0.47–0.74), *p* < 0.001, and HR = 0.74 (0.60–0.92), *p* = 0.006; respectively] when compared to the lowest tertile. Similarly, women with increased levels of physical activity have higher rates of breastfeeding at twelve months, compared to the lowest level [odds ratio OR = 1.71 (95% CI 1.29–2.25) and 1.38 (1.06–1.79)]. Higher levels of physical activity by pregnant women are associated with improved breastfeeding outcomes.

## 1. Introduction

Prenatal physical activity is associated with having a healthy pregnancy, and benefits obstetric and perinatal outcomes, including preventing excessive gestational weight gain, gestational diabetes, gestational hypertension, prenatal depression, and lower rates of instrument delivery [1,2,3,4,5]. The benefits of breastfeeding for both infant and maternal health have been well documented in numerous reviews [6,7,8,9,10].

The US Physical Activity Guidelines Advisory Committee has concluded that during pregnancy there is strong evidence that more physically active women are less likely to gain excessive weight during pregnancy, less likely to develop gestational diabetes or develop postpartum depression than their less active peers [11]. This committee also stated that it was likely that physical activity had no effect on lactation.

As both breastfeeding and physical activity are beneficial it would be reasonable to expect that mothers might want to do both [12]. There is some evidence for associations between physical activity during pregnancy and longer-term postpartum weight loss, lower rates of depression, improved quality of life, and longer breastfeeding [13,14,15]. Although there has been extensive research on the independent benefits of physical activity and breastfeeding for both mothers and infants, the association between these important issues has only rarely been studied [12,15]. Studies have explored the impact of physical activity during lactation on breastfeeding performance or on infant health or growth, but as yet there are no published reports of the association between physical activity during pregnancy and breastfeeding outcomes, including breastfeeding duration [16,17,18,19,20]. During the postpartum period interactions between lactation and physical activity together with diet on maternal weight and/or infant growth have examined [21,22]. Exclusive breastfeeding to around six months results in greater weight reduction than mothers who stopped breastfeeding earlier [22]. There has been one qualitative report of an association between sports women and improved breastfeeding outcomes [23]. Several professional organisations have indicated that postpartum exercise, and occasionally antenatal exercise are compatible with satisfactory lactation, although these are based on expert consensus and not on additional studies [1,24,25,26,27]. We were unable to find published quantitative studies of an association between antenatal exercise and breastfeeding, which resulted in this study.

Vietnam has a population of 95 million and over the past three decades had made great progress in improving overall health, including in reducing infant and maternal mortality [28]. The health system is one of the most efficient in terms of value or the resources used. Almost all deliveries in Vietnam take place within the formal health care system at health centres or district hospitals with referral available to tertiary provincial hospitals [28]. Within the Vietnamese culture, during childbirth and the postpartum period, mothers usually have extensive peer support through her family and community [29]. Breastfeeding initiation is very high, with almost all mothers, (98%) leaving the health care facility breastfeeding their infant [29,30]. However high rates of prelacteal feeding, and the early introduction of complementary foods, results in low rates of exclusive breastfeeding [31,32]. Increasingly infant formula is advertised in Vietnam and its use is increasing, particularly in the cities [33,34]. Most Asian countries, including Vietnam, are experiencing a trend towards increased weight gain in pregnancy, which while still low by Western standards, is associated with increased rates of gestational diabetes [35,36,37]. The objective of this study was to examine associations between physical activity during pregnancy and breastfeeding prevalence at, and breastfeeding cessation before, twelve months postpartum in Vietnamese women.

## 2. Materials and Methods

### 2.1. Design

A multi-centre prospective cohort study was undertaken between August 2015 and December 2017 at six hospitals across three cities in Vietnam [38]. A total of 2030 pregnant women were recruited during their antenatal care visits between 24 and 28 weeks of gestation. The baseline questionnaire completed at recruitment included the Pregnancy Physical Activity Questionnaire. After delivery, participants were followed up at hospital discharge and then at 1, 3, 6 and 12 months postpartum. Detailed information about the recruitment and the catchment area has been presented elsewhere [38]. The study was approved by the Hai Phong University of Medicine and Pharmacy Human Research Ethics Committee (approval no. 05/PHUMPRB) and the Curtin University Human Research Ethics Committee (approval no. HR32/2015).

### 2.2. Participants

Participants were pregnant women who: (1) were permanent residents in the study locations; (2) ≥18 years of age; (3) at 24–28 weeks of gestation; (4) had a singleton pregnancy; (5) did not have any serious pre-existing health conditions (as indicated in medical records); and (6) were able to read the information sheet and sign the consent form. During the course of the study, participants who had serious maternal health problems and, or who were advised not to breastfeed their infants for medical reasons, termination of pregnancy, a still birth, or infant death were excluded from the analysis.

### 2.3. Measurement

The Pregnancy Physical Activity Questionnaire (PPAQ) is a self-reported questionnaire to assess physical activity during pregnancy which has been widely used, including in the Vietnamese context [3,39]. It measures the physical activity of pregnant women during the past three months through 32 activities categorized in four domains including (1) housework/caregiving, (2) occupational, (3) sports/exercise, and (4) commuting. For each activity, duration, frequency and intensity were measured in Metabolic Equivalent Tasks; MET-hours per week [40]. Total physical activity was categorized into four intensity levels: (1) sedentary, (2) light (1.5–<3.0 METs), (3) moderate (3–6 METs), and (4) vigorous (>6 METs) [41]. Energy expenditure of the total physical activity and four domains and intensity levels were reported by tertiles. As few (*n* = 47) women participated in vigorous activity during pregnancy, this variable was categorized into two levels: ‘yes’ and ‘no’.

During pregnancy, healthy women are encouraged to engage in moderate-intensity aerobic activity (at least 150 min per week) as advised by the Department of Health and Human Services (DHHS) and in other guidelines [2,4,11,42]. Physical activity was categorised as ‘yes’ (if they participated 7.5 MET-hours or more per week in sport/exercise activities of moderate-intensity), and ‘no’. In this study the mothers completed the PPAQ at the baseline interview at 24–28 weeks of their pregnancy. After delivery the prevalence of ‘any breastfeeding’ at twelve months postpartum and breastfeeding cessation before twelve months were measured. ‘Any breastfeeding’ is defined as “when a child had received breastmilk (direct from the breast or expressed or stored breastmilk) with or without other drink, formula or other infant food” [43]. Infant feeding was assessed in face-to-face interviews through the question *“How are you feeding your baby?”* at hospital discharge and at 1, 3, 6 and 12 months. Breastfeeding duration was recorded in weeks by asking *“How old was your baby when you stopped breastfeeding?”*.

Factors to be considered as confounders were determined from the literature, predominantly from studies in Vietnam, Japan and in neighboring China [16,29,44,45,46,47,48]. These included maternal characteristics such as maternal age (years) (<25; 25–35; >35), occupation (currently working; currently not working), education level (secondary school or lower; high school; diploma/university or above), parity (0; 1; ≥2), pre-pregnancy body mass index (kg/m^2^) (overweight ≥ 23; not overweight < 23), which were collected at the baseline interview. Gestational diabetes was diagnosed between 24 and 28 weeks of gestation following the criteria from the International Association of the Diabetes and Pregnancy Study Groups, with at least one glucose value equal or above the threshold: fasting plasma glucose ≥ 5.1 mmol/L, 1-h plasma glucose ≥ 10.0 mmol/L, 2-h plasma glucose ≥ 8.5 mmol/L [49]. Maternal and neonatal variables were obtained from medical records at hospital discharge including gestational age (preterm < 37 weeks; not preterm ≥ 37 weeks), caesarean section (yes; no), low birth weight (yes < 2500 g; no ≥ 2500 g), and admission to neonatal intensive care unit (yes; no).

### 2.4. Data Analysis

Descriptive statistics and group comparisons between participants’ breastfeeding and not breastfeeding at twelve months were made, using chi-square test or Fisher exact test. Logistic regression analyses were then performed to determine the associations between physical activity and breastfeeding prevalence at twelve months, with adjusted odds ratio (OR) and associated 95% confidence intervals (CI) to account for the effects of plausible confounding factors. Cox’s regression model with adjusted hazard ratios (HR) and associated 95% CIs were used to report the effect of physical activity on the risk of breastfeeding cessation. Covariates were maternal age, occupation, education level, parity, pre-pregnancy body mass index, gestational diabetes status, gestational age, caesarean section, low birth weight, and admission to neonatal intensive care unit. All statistical analyses were undertaken using the SPSS package version 22 (IBM, Armonk, NY, USA).

## 3. Results

A total of 2030 pregnant women were recruited at baseline. Then 19 women were excluded; eight mothers endured a still birth, two had human immunodeficiency virus infection, one terminated her pregnancy due to intrauterine growth restriction, seven infants died within 12 months, and one infant was transferred to an orphanage. Another 296 women declined to participate or were lost to follow-up during the 12-month postnatal period. Therefore, 1715 participants remained at the 12-month survey, yielding a participation rate of 85%. There were no significant differences in age, parity and other demographic characteristics between the ‘loss-to-follow-up’ group and the completed group, except small differences in occupation and education level (*p* < 0.05).

Table 1 describes the demographic, maternal and neonatal characteristics of participants who were breastfeeding (*n* = 1232) and not breastfeeding at twelve months (*n* = 483), which makes a total of 1715 mother-infant dyads included in the analysis. Women who maintained breastfeeding to twelve months or more were likely to have a higher education level (*p* < 0.001), less overweight (*p* < 0.001), and less gestational diabetes (*p* = 0.001) compared to those who had already stopped breastfeeding.

Table 2 explores the association between the levels and types of physical activity intensity during pregnancy and breastfeeding status at twelve months. As can be seen from Table 2, those who had a higher level of physical activity were more likely to be breastfeeding at 12 months (*p*-value < 0.05). This finding was observed in all physical activity related variables including total physical activity, eight sub-groups of intensities and domains. However, after adjustments for all confounders, significant differences were not found for the ‘vigorous’ and ‘occupational’ activities. See detailed adjusted ORs in Table 2.

**Figure 1:**
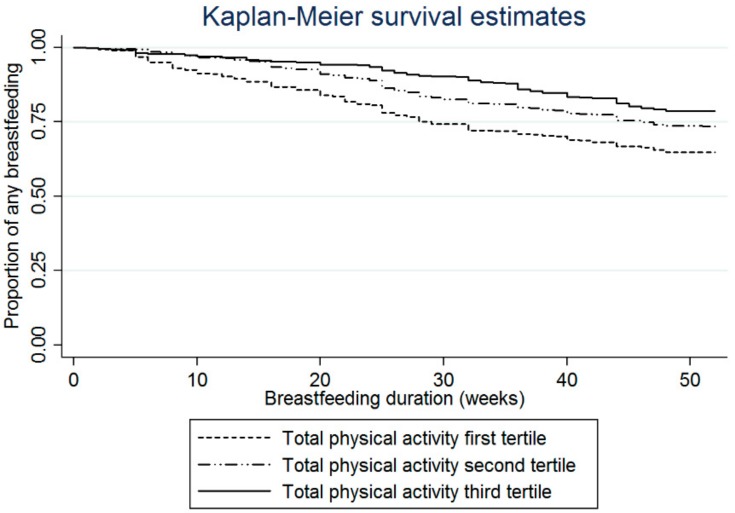
Kaplan-Meier curves of ‘any breastfeeding’ in the first twelve months postpartum for mothers with three different levels of physical activity (tertiles) during pregnancy (log-rank test *p* < 0.001).

**Figure 2:**
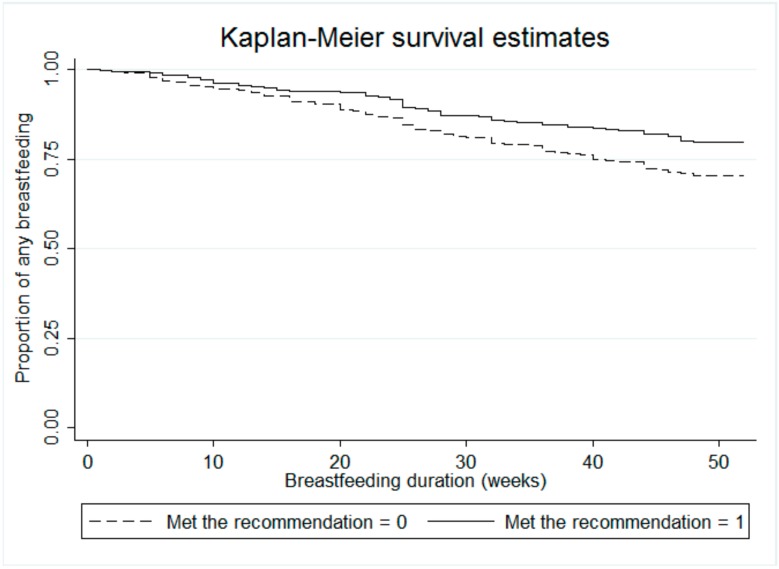
Kaplan-Meier curves of ‘any breastfeeding’ in the first twelve months postpartum for mothers who met and not meet the recommendation of physical activity during pregnancy (log-rank test *p* = 0.0005). The cutoff point for physical activity used the DHHS Guidelines [4].

An assumption of the models in Figure 1 and Figure 2 and Table 3 is that all women starting breastfeeding at the same point after delivery (week zero) and the actual rate of breastfeeding within one week was very close to this at 98.4%. Results from the rank sum tests show significant differences in any breastfeeding duration between the two groups of women: those who had lower total physical activity during pregnancy, and who did not meet the physical activity guideline had a higher risk of stopping breastfeeding compared to active women (*p* < 0.001 and *p* = 0.0005 respectively). The Kaplan-Meier curves in Figure 1 and Figure 2 represent the difference in breastfeeding duration between active and inactive women (by total physical activity, Figure 1, and by meeting the recommendations for physical activity Figure 2).

The adjusted hazard ratios showing a significant association between prenatal physical activity on ceasing breastfeeding are presented in Table 3, also except for the vigorous and occupational activities.

## 4. Discussion

The current findings indicate that women who undertake physical activity during pregnancy are less likely to terminate breastfeeding at any point up to twelve months, and have a higher rate of breastfeeding at twelve months. The rate of breastfeeding at 12 months was 71.8%, which is similar to or just above other reported rates in Asia [50]. In China and Japan breastfeeding rates for the first six months are similar to Vietnam, but between 6 and 12 months rates in Vietnam are maintained at higher rate [48,51,52,53] The findings from this study in Vietnam related to physical activity, provide further evidence to strengthen current guidelines showing that physical activity during pregnancy appears to have no adverse effects on breastfeeding [1,4,5,42].

A controlled trial of thirty-three mothers from six to eight weeks postpartum found aerobic exercise found no association between exercise and breastmilk volume and composition (and maternal prolactin levels), but improved oxygen uptake by the mothers [17]. A cohort study in Australia found that the intensity levels of postnatal exercise had no association with the duration of ‘any breastfeeding’ up to twelve months, and the duration of full breastfeeding to six months postpartum [16]. While several studies have examined the impact of physical activity after birth on breastfeeding, this is the first published study examining the association of all aspects (total physical activity, four sub-groups of intensity and four domains, and compliance to the guideline of DHHS) of prenatal physical activity and long-term breastfeeding. Previous studies have found no association between postnatal physical activity and lactation. However, this study suggests that prenatal physical activity is beneficial to breastfeeding outcomes. The possible causes of breastfeeding [15,17]. cessation among less active women include an unhealthy lifestyle, and a lack of knowledge and research about the benefits of both physical activity and breastfeeding.

Population lifestyles include patterns of health behaviours that cluster together, positive and negative are a well-known phenomenon in health promotion studies [54,55]. In intervention trials and subsequent programs it is best practice to address a number of lifestyle factors together, resulting in a greater improvement in health outcomes [56]. Many multiple risk factor interventions have demonstrated the efficiency of this approach [57]. However, the clustering of behaviours means that it is not always possible to adjust for all potential confounding factors and covariates. This is a limitation of cohort studies, including this one.

The strengths of this current study are that it is a multicenter longitudinal study with a large sample size, included prospective measurements of breastfeeding cessation (weeks) from discharge to twelve months and appropriate statistical methods were used to account for interactions between covariates. Physical activity guidelines and recommendations should not only be focused on perinatal period and delivery outcomes, but should also include breastfeeding benefits [1,4,5,42]. The main finding from this study is evidence for the beneficial role of physical activity during pregnancy and in the postpartum periods [4,5,58].

There are several limitations to be considered when interpreting the results of this study. Although participants were recruited from six hospitals of three cities, the sample may not be representative of the all parts of Vietnam. Although the physical activity was self-reported, the questionnaire has been widely used worldwide and has been validated for Vietnamese women [39]. Self-reported physical activity has been widely used in epidemiological studies with good results [13]. Only a few women reached high levels of physical activity and the results may not apply to these high levels, but very high levels of activity are not recommended in pregnancy [1]. This study only focused on confounding factors related to maternal and birth outcomes, but not social, parental factors, professional support as well as physical activity after postpartum which can affect the breastfeeding outcomes. Although postpartum physical activity could be a modifying factor for breastfeeding outcomes, the same physical activity questionnaire could not be used for the postnatal period and was not included in this study. Future studies should include measurement of antenatal and postnatal exercise in a longitudinal study.

## 5. Conclusions

Women who are physically active during the lactation period are known to have better breastfeeding outcomes. In this prospective cohort study of Vietnamese women, it was found that higher levels of physical activity during pregnancy are associated with an increased likelihood of breastfeeding at 12 months postpartum. Given the well-recognized benefits of breastfeeding and physical activity for both mother and child, midwifery nurses and medical staff should be aware that this association may enhance breastfeeding. A trial of health promotion to encourage physical activity for pregnant women may prove to be beneficial to mother and infant.

## Figures and Tables

**Table 1:** Characteristics of participants by breastfeeding at 12 months (*N* = 1715).

Variables *N* (%)	Overall	Any Breastfeeding at 12 Months		*p* ^a^
	*N* (%)	Yes	No	
Total	1715 (100)	1232 (71.8)	483 (28.2)	
Maternal age (years)				0.116
<25	548 (32.0)	380 (30.9)	168 (34.8)	
>35	1167 (68.0)	852 (69.1)	315 (65.2)	
Occupation				0.374
Currently not working	535 (31.2)	392 (31.8)	143 (29.6)	
Currently working	1180 (68.8)	840 (68.2)	340 (70.4)	
Education level				**<0.001**
Secondary school or lower	576 (33.6)	375 (30.4)	201 (41.6)	
High school	447 (26.1)	323 (26.2)	124 (25.7)	
Diploma/university or above	692 (40.3)	534 (43.4)	158 (32.7)	
Parity				0.059
0	658 (38.4)	464 (37.7)	194 (40.2)	
1	638 (37.2)	448 (36.3)	190 (39.3)	
≥2	419 (24.4)	320 (26.0)	99 (20.5)	
Pre-pregnancy body mass index ^b^				**<0.001**
Overweight: ≥23	189 (11.0)	113 (9.2)	76 (15.7)	
Not overweight: <23	1526 (89.0)	1119 (90.8)	407 (84.3)	
Gestational diabetes ^c^				**0.001**
Yes	373 (21.8)	243 (19.8)	130 (27.0)	
No	1336 (78.2)	985 (80.2)	651 (73.0)	
Gestational age (weeks)				0.281
Preterm: <37	71 (4.1)	47 (3.8)	24 (5.0)	
Not preterm: ≥37	1644 (95.9)	1185 (96.2)	459 (95.0)	
Caesarean section				0.232
Yes	654 (38.1)	459 (37.3)	195 (40.4)	
No	1061 (61.9)	773 (62.7)	288 (59.6)	
Low birth weight (g)				0.159
Yes: <2500	64 (3.7)	41 (3.3)	23 (4.8)	
No: ≥2500	1651 (96.3)	1191 (96.7)	460 (95.2)	
Admission to neonatal intensive care unit				0.376
Yes	44 (2.6)	29 (2.3)	15 (3.1)	
No	1671 (97.4)	1203 (97.7)	468 (96.9)	

^a^ From Chi-square test or Fisher exact test; ^b^ Classified for Asian populations according to World Health Organization; ^c^ Based on International Association of the Diabetes and Pregnancy Study Groups criteria 2010 [49]. Bolded *p*-values are significant.

**Table 2:** Associations of levels of physical activity intensity and types during pregnancy with reported breastfeeding at 12 months among Vietnamese women (*N* = 1715).

Physical Activity Variables	Any Breastfeeding at 12 Months		Adjusted ^a^ OR 95% CI
	Yes	No	
**Total PA (METs h/week)**			
Mean, SD (125.0, 57.6)	129.7 (59.0)	112.9 (52.1)	
1st tertile (8.4–<94.3)	368 (64.3)	204 (35.7)	1.00
2nd tertile (94.3–<144.2)	419 (73.2)	153 (26.8)	**1.38 (1.06–1.79)**
3rd tertile (144.2–362.8)	445 (77.9)	126 (22.1)	**1.71 (1.29–2.25)**
**Domain of activity**			
**Household/caregiving (METs h/week)**			
Median, IQR (50.1, 52.0)	53.4 (55.3)	42.7 (42.7)	
1st tertile (0–<37.3)	369 (64.1)	207 (35.9)	1.00
2nd tertile (37.3–<68.4)	412 (72.5)	156 (27.5)	**1.42 (1.09–1.85)**
3rd tertile (68.4–231.0)	451 (79.0)	120 (21.0)	**1.85 (1.39–2.47)**
**Occupational (METs h/week)**			
Median, IQR (33.6, 45.1)	33.6 (46.2)	33.6 (45.1)	
1st tertile (0–<10.7)	409 (71.2)	165 (28.8)	1.00
2nd tertile (10.7–<41.2)	400 (70.0)	171 (30.0)	1.04 (0.78–1.39)
3rd tertile (41.2–176.2)	423 (74.2)	147 (25.8)	1.23 (0.91–1.66)
**Sports/exercise (METs h/week)**			
Median, IQR (5.6, 5.6)	5.6 (5.6)	0.0 (5.6)	
1st tertile (0–0)	524 (67.4)	253 (32.6)	1.00
2nd tertile (0–<5.6)	422 (73.1)	155 (26.9)	1.20 (0.94–1.54)
3rd tertile (5.6–81.4)	286 (79.2)	75 (20.8)	**1.76 (1.30–2.39)**
**Commuting (METs h/week)**			
Median, IQR (7.9, 13.1)	7.9 (13.1)	7.0 (11.4)	
1st tertile (0–<4.4)	377 (65.6)	198 (34.4)	1.00
2nd tertile (4.4–<12.3)	435 (73.6)	156 (26.4)	**1.35 (1.04–1.76)**
3rd tertile (12.3–170.6)	420 (76.5)	129 (23.5)	**1.59 (1.21–2.08)**
**Sedentary (h/week)**			
Median, IQR (38.0, 33.6)	38.0 (34.1)	39.4 (32.2)	
1st tertile (0–<23.1)	432 (75.4)	141 (24.6)	1.00
2nd tertile (23.1–<46.9)	395 (68.9)	178 (31.1)	**0.75 (0.58–0.99)**
3rd tertile (46.9–110.1)	405 (71.2)	164 (28.8)	**0.74 (0.55–0.99)**
**Light (METs h/week)**			
Median, IQR (52.0, 46.2)	53.9 (46.9)	47.3 (42.5)	
1st tertile (0–<38.9)	387 (67.5)	186 (32.5)	1.00
2nd tertile (38.9–<69.3)	403 (70.3)	170 (29.7)	1.03 (0.80–1.34)
3rd tertile (69.3–166.8)	442 (77.7)	127 (22.3)	**1.51 (1.14–2.00)**
**Moderate (METs h/week)**			
Median, IQR (18.2, 36.4)	23.1 (37.1)	12.3 (23.1)	
1st tertile (0–<10.9)	371 (61.4)	233 (38.6)	1.00
2nd tertile (10.9–<31.9)	405 (73.8)	144 (26.2)	**1.63 (1.25–2.11)**
3rd tertile (31.9–203.5)	456 (81.1)	106 (18.9)	**2.47 (1.86–3.27)**
**Vigorous (METs h/week) (*n*; %)**			
Yes (MET-h/week>0) (1668; 97.3)	34 (72.3)	13 (27.7)	1.16 (0.59–2.26)
No (47; 2.7)	1198 (71.8)	470 (28.2)	1.00
**Met exercise guideline (*n*; %)**			
Yes ^b^ (359; 20.9)	285 (79.4)	74 (20.6)	**1.65 (1.23–2.20)**
No (1356; 79.1)	947 (69.8)	409 (30.2)	1.00

^a^ Adjusted for maternal age (<25; 25–35; >35), occupation (currently not working; currently working), education level (secondary school or lower; high school graduate; college/university or above), parity (0; 1; ≥2), pre-pregnancy body mass index (overweight; not overweight), gestational diabetes (yes; no), gestational age (preterm; not preterm), caesarean section (yes; no), low birth weight (yes; no), and admission to neonatal intensive care unit (yes; no); ^b^ Meeting Department of Health and Human Services guidelines of > 7.5 MET h/week in sports/exercise activities of moderate-intensity or greater [4]; Abbreviations: OR, Odds ratio; CI, Confident Interval; MET, Metabolic Equivalent of Task; SD, Standard Deviation; IQR, Interquartile Range. Bolded odds ratio values are significant.

**Table 3:** Cox regression models of any breastfeeding duration and physical activities among Vietnamese women (*N* = 1715).

Physical Activity Variables	Adjusted ^a^ HR 95% CI	*p*-Value
**Total PA (METs h/week)**		
1st tertile	1.00	
2nd tertile	0.74 (0.60–0.92)	**0.006**
3rd tertile	0.59 (0.47–0.75)	**<0.001**
**Domain of activity** **Household/caregiving (METs h/week)**		
1st tertile	1.00	
2nd tertile	0.73 (0.59–0.91)	**0.005**
3rd tertile	0.57 (0.43–0.73)	**<0.001**
**Occupational (METs h/week)**		
1st tertile	1.00	
2nd tertile	0.94 (0.74–1.19)	0.627
3rd tertile	0.80 (0.62–1.03)	0.082
**Sports/exercise (METs h/week)**		
1st tertile	1.00	
2nd tertile	0.85 (0.69–1.04)	0.116
3rd tertile	0.63 (0.48–0.82)	**0.001**
**Commuting (METs h/week)**		
1st tertile	1.00	
2nd tertile	0.77 (0.62–0.96)	**0.020**
3rd tertile	0.66 (0.53–0.83)	**<0.001**
**Intensity**		
**Sedentary (h/week)**		
1st tertile	1.00	
2nd tertile	1.27 (1.01–1.59)	**0.042**
3rd tertile	1.29 (1.01–1.64)	**0.044**
**Light (METs h/week)**		
1st tertile	1.00	
2nd tertile	0.96 (0.77–1.19)	0.704
3rd tertile	0.68 (0.54–0.87)	**0.002**
**Moderate (METs h/week)**		
1st tertile	1.00	
2nd tertile	0.67 (0.54–0.83)	**<0.001**
3rd tertile	0.45 (0.35–0.57)	**<0.001**
**Vigorous (METs h/week)**		
Yes (MET-h/week > 0)	0.99 (0.57–1.74)	0.981
**Met exercise guideline**		
Yes ^b^	0.67 (0.52–0.86)	**0.002**
No	1.00	

^a^ Adjusted for maternal age (<25; 25–35; >35), occupation (currently not working; currently working), education level (secondary school or lower; high school graduate; college/university or above), parity (0; 1; ≥2), pre-pregnancy body mass index (overweight; not overweight), gestational diabetes (yes; no), gestational age (preterm; not preterm), caesarean section (yes; no), low birth weight (yes; no), and admission to neonatal intensive care unit (yes; no); ^b^ Meeting Department of Health and Human Services guideline of > 7.5 MET h/week in sports/exercise activities of moderate-intensity or greater [4]; Abbreviations: HR, Hazard Ratio; CI, Confident Interval; MET, Metabolic Equivalent Task. Bolded *p*-values are significant.
